# Embodied carbon dataset for residential building structures in Finland

**DOI:** 10.1016/j.dib.2026.113243

**Published:** 2026-09-05

**Authors:** Chloe Kiernicki, Mario Kolkwitz, Tapio Kaasalainen, Satu Huuhka

**Affiliations:** Tampere University, School of Architecture, P. O. Box 600, FI-33014 Tampereen yliopisto, Finland

**Keywords:** Building stock, Construction materials related CO_2_ emissions, Domestic buildings, Embodied CO_2_, Housing stock, Life cycle analysis (LCA), Material stock and flow analysis (MSFA), Product stage

## Abstract

This dataset contains estimates of embodied carbon in building structures for a total of 53 residential building cohorts in Finland from the 1940s to the 2010s, which have been reconstructed using present-day GWP coefficients. The building types covered are one dwelling houses and blocks of flats. The dataset was created by combining and extending two existing datasets: (1) a building part specific material inventory dataset for residential buildings in Finland, and (2) the generic Finnish national emissions database CO2data. Materials presented in the former dataset were matched with the most appropriate corresponding material in the latter. The volumes and densities of materials were multiplied by the GWP coefficients of present-day Finnish construction materials to approximate the embodied carbon of materials present in structures of Finnish housing archetypes. The resulting embodied carbon estimates are presented at the scale of a building, a horizontal building level, and at smaller scales of components. The embodied carbon estimates are also presented as intensities that divide by the floor area of building archetypes; this enables comparing structural embodied carbon between archetypes. The data can be used for life cycle analysis (LCA) on the neighbourhood level or higher, or combined with the original material inventory dataset, for a combined material stock and flow analysis (MSFA) and LCA.

Specifications Table

**Table:** 

Subject	Engineering & Materials science
Specific subject area	Combined material stock and flow analysis (MSFA) and life cycle analysis (LCA) of buildings that need embodied carbon data.
Type of data	Table Processed
Data collection	The dataset was created by transforming an existing material inventory dataset of Finnish residential building structures into an embodied carbon dataset. This was achieved by associating materials in the material inventory dataset with present-day GWP coefficients for embodied carbon drawn from the Finnish national emissions database, which includes generic values that represent construction products typically used in Finland today.
Data source location	FinlandThis original dataset was created by combining and extending two existing datasets: • Kaasalainen, T., Kolkwitz, M., Nasiri, B., Huuhka, S. & Hughes, M. (2026). Building part specific material inventory dataset for residential buildings in Finland. Zenodo. https://doi.org/10.5281/zenodo.22043053 ○ Version: 1.0.5 Country: Finland City: Vantaa, latitude 60.29, longitude 25.04 Original data sources: ▪ Lupapiste Kauppa, https://kauppa.lupapiste.fi/ ▪ National Archives of Finland, https://astia.narc.fi/ ▪ City of Tampere Building Control Services archive • Finnish Environment Institute. (2026). National Emissions Database CO2data. Building Construction Emissions Database. https://CO2data/rakentaminen/#en ○ Version: 1.01.009 Country: Finland
Data accessibility	Repository name: Zenodo Data identification number: 10.5281/zenodo.21024219 Direct URL to data: https://doi.org/10.5281/zenodo.22206193
Related research article	None

## 1 Value of the Data

• Embodied carbon estimates of building structures in archetypal buildings can help to quantify and understand the carbon embodied in existing building stocks. Combined with data on buildings’ operational carbon emissions, such data can help to manage the climate impacts of spatial planning strategies, such as building replacement or infill development, on different spatial scales, from the neighbourhood level to the national level.
• Estimating the embodied carbon intensity enables the comparison of embodied carbon across archetypes and across buildings with varying floor areas.
• For the contemporary stock, the dataset includes embodied carbon estimates of 16 contemporary residential building archetypes (8 apartment buildings and 8 houses) from the 2000s and the 2010s. When project-specific data is not available, this data can be used in lieu of it in building and urban LCA.
• For the historic stock, the dataset includes embodied carbon estimates of 37 residential building archetypes built between the 1940s and 1990s, which have been reconstructed using present-day GWP coefficients. Since the inventory is provided on a building part level, it can be used for estimating embodied carbon impacts of renovation measures by only including the relevant parts and the extent of their renovation.
• The reconstructed embodied carbon estimates of historic building archetypes can also be used in life cycle analysis frameworks where embodied carbon is allocated to present or future users, e.g. when multiple life cycles are considered, like in component reuse. However, this application demands caution: embodied carbon emissions occurred when the building materials were originally manufactured, and no allocation could reverse this.
• The data is useful for extending material stock and flow analysis (MSFA) towards LCA. In addition to research, this data can be used for practice-based analysis in urban planning and related decision-making on municipal to national level.

## 2 Background

The dataset was created to facilitate construction materials related life cycle analysis (LCA) on the urban scale in Finland, as well as combined material stock and flow analysis (MSFA) and LCA. City information models (CIMs) or urban digital twins (UDTs) can be used for spatial greenhouse gas (GHG) accounting on the subnational level, but they rely on the availability of granular bottom-up data, like carbon footprint assessments of buildings [1]. The current dataset [2] provides such data on the estimated embodied carbon of archetypal Finnish residential building structures from different decades. This type of data is still largely omitted from urban spatial GHG accounting, as UDTs’ present focus lies primarily on buildings’ operational energy use [1]. It is, however, a prerequisite for considering carbon more holistically in such accounting. In the future, such data is expected to become increasingly available from mandatory climate declarations of new buildings and from the integration of such buildings’ building information models (BIM) and CIMs/UDTs [1]. The archetype-based embodied carbon estimates of the current dataset can be used in the meantime. Moreover, these new developments only concern new buildings. When embodied carbon data is needed for existing buildings, the estimates in this dataset can be utilised.

## 3 Data Description

This article describes the embodied carbon dataset that can be found at [2]. The dataset contains embodied carbon estimates of building structures in archetypes of Finnish residential buildings, reconstructed using present-day GWP coefficients and material inventories of historical buildings. The included residential buildings are divided into two housing typologies: blocks of flats and one dwelling houses. The archetypes represent these typologies built at decadal intervals from the 1940s through the 2010s. The dataset reconstructs the amount of carbon emitted during the construction of the archetypes’ structures, based on present-day carbon emissions data from the Finnish generic national emissions database, CO2data [3]. When this manuscript refers to embodied carbon values or intensities in the dataset [2], it always means estimates that are based on present-day carbon emissions data and material inventories of historical and contemporary buildings.

Because the embodied carbon dataset is underlain by the material data presented in [4] and described in [5], the structure is informed by the existing dataset. The OpenDocument Spreadsheet (ODS) file is divided into the following sheets: *description, data_building, data_building_level, data_building_part, data_building_subpart, reference_co2data, reference_coefficients*, and *reference_grades*. Each data sheet contains material-specific volumes (m^3^), embodied carbon (kgCO_2_-eq), and embodied carbon intensity (kgCO_2_-eq/floor area), in addition to the basic characteristics of the corresponding archetypes. Table 1 presents this information and the nomenclature of column titles in the dataset [2].

**Table 1:** Column IDs in the dataset [2]. Based on the structure, and therefore compatible with, the structure of dataset [4].

Column ID	Description
b_id	Building specific identifier code comprising the following: abbreviated building type (BOF for blocks of flats, ODH for one dwelling houses), initial letters for main bearing and façade materials (brick, concrete, or wood; e.g. CB for bearing concrete with brick façade), cohort decade (e.g. 1960), and a running number. For buildings without separate bearing and façade materials (e.g. massive brick structures) a single initial is used for the material.
b_type	Type of housing, either a block of flats or one dwelling house.
b_completionyear	The year when the building finished construction.
b_structuralsystem	The load-bearing system of the building, currently either massive clay bricks, precast concrete panels, insulated cavity brick walls, aerated concrete blocks, balloon frame, concrete exterior walls, or solid logs.
b_bearingmaterial	The primary material in the load-bearing structure, either concrete, brick, or wood.
b_facadematerial	The primary material used in the façade, either concrete, brick, or wood.
b_storeys	The number of storeys, including attic [+a] and/or basement [+b].
b_gfa	The gross floor area (GFA) of the building, following the Finnish definition that does not include spaces that do not support the central program of housing. Reported in m^2^.
b_tfa	The total floor area (TFA) of the building, following the Finnish definition and including spaces in attics and basements excluded from GFA. Reported in m^2^.
bl_height	The height of a horizontal level in the building. Reported in m.
bl_gfa	The GFA of one building level. Reported in m^2^.
bl_tfa	The TFA of one building level. Reported in m^2^.
bp_level	A horizontal layer of a building, described in a row.
bp_part	A part of a level, described in a row.
bp_subpart	A subpart of a part, described in a row.
ei_gfa	Total embodied carbon intensity of the materials in one row. Reported in kgCO_2_-eq/m^2^ GFA.
ei_tfa	Total embodied carbon intensity of the materials in one row. Reported in kgCO_2_-eq/m^2^ TFA.
ei_s_gfa_[material]	Material-specific embodied carbon intensity of respective structural materials in one row. Reported in kgCO_2_-eq/m^2^ GFA.
ei_c_gfa_[material]	Material-specific embodied carbon intensity of respective complementary materials. Reported in kgCO_2_-eq/m^2^ GFA.
ei_s_tfa_[material]	Material-specific embodied carbon intensity of respective structural materials. Reported in kgCO_2_-eq/m^2^ TFA.
ei_c_tfa_[material]	Material-specific embodied carbon intensity of respective complementary materials. Reported in kgCO_2_-eq/m^2^ TFA.
v_s_[material]	The volume of respective structural materials in a row. Reported in m^3^.
v_c_[material]	The volume of respective complementary materials in a row. Reported in m^3^.
e_s_[material]	The embodied carbon of respective structural materials. Reported in kgCO_2_-eq.
e_c_[material]	The embodied carbon of respective complementary building components. Reported in kgCO_2_-eq.

**Fig. 1:**
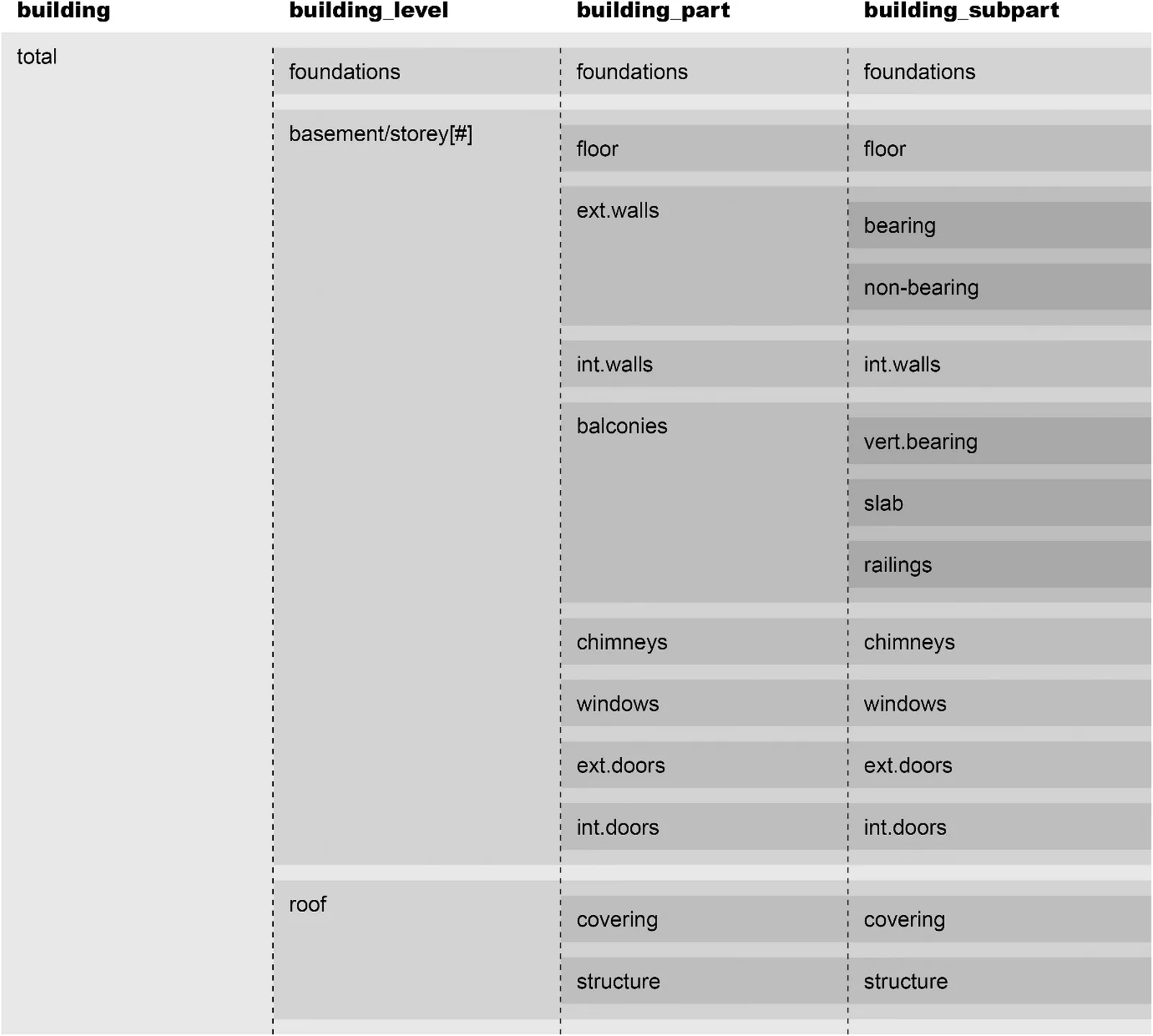
Relationship between *building, building_level, building_part*, and *building_subpart*. Published with the updated material inventory dataset [4].

The embodied carbon estimate of each building archetype is composed of four hierarchical degrees of aggregation: the building, the level, the part, and the subpart. The *data_building* sheet describes the estimated embodied carbon of 53 building archetypes at the building scale, with each row describing one archetype and each archetype being fully described by one row. The *data_building_level* sheet describes the embodied carbon estimate of one vertical level of an archetype per row: if a building has five storeys, then there is a row for each storey, as well as one each for the foundation, roof, and, if present, the basement. The *data_building_part* sheet distinguishes the embodied carbon estimates between elements such as windows, walls, and balconies. Finally, within specific elements, such as exterior walls, further divisions are made to clarify embodied carbon estimates in bearing versus non-bearing structures; this information is documented in the *data_building_subpart* sheet. The volumes of embodied carbon estimates in subparts within one part sum to the value of the total part; the volumes of parts within one level sum to the value of the total level; the volumes of levels within one building sum to the value of the total building structure. The relationship between the sheets is depicted in Fig. 1.

Each building hierarchy is divided into *building structure* and *complementary building components*. Complementary building components currently refer to windows and doors, while materials classified as part of the building structure are found in all other elements. Complementary building components are separated to allow for future updates to the datasets [2] and [4] that include other types of complementary components, like mechanical, electrical and plumbing (MEP) components, elevators, etc.

The *reference_co2data* sheet contains present-day CO_2_ emissions factors, drawn from the Finnish generic national emissions dataset [3], for the materials present. The factors represent current fossil emissions for LCA modules A1–A3 [6] measured in kilograms of CO_2_ equivalents (kgCO_2_-eq) per kilogram of material. Both Conservative Global Warming Potential (GWP) and Typical GWP, provided by the emissions dataset [3], are included in the sheet. Typical GWP has been determined based on Finnish and regional Environmental Product Declarations (EPDs), and Conservative GWP has been estimated by multiplying the Typical GWP by a conservative factor of 1.2, which is consistent across material types [7]. The purpose of the factor is to account for high variation in underlying EPD data and to therefore ensure embodied carbon is not underestimated [7]. To follow Finnish climate declaration policy requirements [8], the current dataset [2] uses Conservative GWP. However, users can choose to utilise Typical GWP values if local requirements differ. In addition, the sheet contains the densities used for the respective materials, as well as references to the documentation from the Finnish generic national emissions dataset [3]. The *reference_coefficients* sheet translates material Conservative GWP values from the *reference_co2data* sheet to the corresponding building subpart. The *reference_grades* sheet provides the stratification of concrete, steel rebar, and brick ties found in the housing archetypes. The material grades are specific to the legislation of the decade of construction, the component in the structure, and/or market availability of products, as explained in greater detail in the section ‘Material Stratification’. The grades correspond to different GWP values, so the stratification provides greater granularity.

The *description* sheet describes the column IDs presented in Table 1, which are the headers used across the data sheets. Concise information about the materials identified in the building archetypes is provided, as well as definitions of the building parts and subparts detailed above.

The dataset contains two ODS files: *embodied carbon dataset.ods* and *static embodied carbon dataset.ods.* The former file contains the formulas that determine the embodied carbon values, while the latter file contains only the values. This additional formula file allows interested users to investigate the aggregation of the estimated values and, for example, apply alternative GWP coefficients. Should the user have no such needs, the use of the static dataset file is recommended, as to not inadvertently compromise the aggregation due to e.g. differences in software function. Conversely, the static dataset file can be referenced to ensure this has not happened.

## 4 Experimental Design, Materials and Methods

The embodied carbon dataset [2] was created by combining and extending two existing open access datasets: the updated building part specific material inventory (MI) dataset for residential buildings in Finland [4], the first version of which is described in [5], and the Finnish generic national emissions database CO2data [3]. Because the MI dataset [4] only contains materials in building structures and excludes materials in MEP components, the embodied carbon dataset [2] has a similar scope.

To estimate the embodied carbon based on present-day carbon intensities and produce the current dataset [2], each material present in the MI dataset [4] was matched to a corresponding material present in the CO2data dataset [3], as detailed in the subsection ‘Material Matching’. Following this, as detailed in the subsection ‘Estimating Embodied Carbon Emissions’, the volume and density of every material present in each subpart were multiplied by the material’s respective Conservative GWP coefficient. The resulting carbon estimates were then summed to form the different levels of aggregation and, finally, divided by floor area to produce embodied carbon intensities. The last subsection ‘Quality Control’ describes the measures taken to assure the quality of the resulting dataset [2].

### 4.1 Data provenance

This embodied carbon dataset [2] is grounded in the updated MI dataset [4], which is the largest and most comprehensive of its kind for Finland. It follows a similar structure, and it utilises the published archetypes and material volumes presented in [4]. The shared structure supports interoperability between the datasets and, correspondingly, concurrent updates.

The archetypes represent blocks of flats and one dwelling houses in Finland, built between the 1940s and 2010s. The underlying building structures are described in greater detail in the updated MI dataset [4] and the related article [5]. The material volumes were derived from construction drawings and models [5]. To facilitate adjustments to cohort, building part, or/and material-specific densities, material volumes from [4] are also included in the current dataset [2]. Furthermore, although the MI dataset contains material masses as the product of volume and density, in the dataset [2], material volumes were used as a primary form of data.

The CO2data dataset [3] was created and is maintained by the Finnish Environment Institute with a mandate from the Finnish Ministry of the Environment to support legally required climate declarations of buildings [7,8]. It contains generic GWP coefficients for present-day embodied carbon of building materials and products typically used in Finland. The coefficients estimate LCA modules A1–A3 [6], which quantify the emissions associated with the extraction of natural resources, transport to manufacturing facilities, and the production of the materials present. This generalised data can be used when the specific products, their manufacturers, and embodied carbon contents are unknown. These generic coefficients are based on actual products’ EPDs [7], as detailed in the background reports on each product page in the database.

### 4.2 Material matching

The material matches are presented in Table 2, which displays the building structure materials, and Table 3, which displays the complementary materials.

**Table 2:** Material matching between the building structure materials in the MI dataset [4] and CO2data [3] for all materials present in dataset [2].

MI dataset material	Material matching criteria	CO2data material match	Match quality	Notes
Aerated concrete (loose)	Loose aerated concrete not available in CO2data, so solid material used.	Autoclaved aerated concrete block, interior walls	2	In the current MI dataset, loose aerated concrete is only located in building interiors.
Aerated concrete (solid)	Material located in *ext.walls*.	Autoclaved aerated concrete block, exterior walls	1	
	Material located in *int.walls*.	Autoclaved aerated concrete block, interior walls	1	
Aluminium	Used in *balconies_railing* subpart in post-1990s building archetypes.	Aluminium profile, scrap 100%	1 / 2 (depending on the scrap content)	See Table 3. May underestimate carbon emissions if the actual scrap content is lower.
Bitumen felt	Single-ply roofing, found in building archetypes *ODH_B[X]_1960_1, ODH_W[X]_1960_1, ODH_W[X]_1970_1.*	Bitumen waterproofing membrane, single-ply roofing system, TL1	1	In the current MI dataset, bitumen felt is only located in *roof_structure* subpart.
	Double-ply roofing, found in building archetypes BOF_C[X]_1970_1, *BOF_C[X]_2000_1,* *BOF_C[X]_2010_1,* *ODH_B[X]_1970_1.*	Bitumen waterproofing membrane, bottom layer, TL2/TL3 + Bitumen waterproofing membrane, top layer membrane, TL2, applied as one GWP value based on the ratio of membrane thicknesses (bottom layer 3.6 mm, top layer 3 mm)	1	
Brick	Red brick assumed as the most common type to the alternatives available in CO2data (light brick, calcium silicate brick).	Brick, red	1/2 (archetype-dependent)	If the user knows the brick type in their case, they can adjust the coefficient.
Brick (sawdust)	Sawdust bricks not available in CO2data, so regular red brick used.	Brick, red (density adjusted)	2	Historic product.
Concrete	Material located in *roof_covering.*	Concrete roofing tile	1	
	Material located everywhere else.	See Table 4	1 / 2 (see Table 4)	
Expanded clay (loose)	The only type of loose expanded clay available in CO2data.	Expanded clay crushed aggregate	1	
Expanded clay (solid)	The only type of solid expanded clay available in CO2data.	Concrete block, lightweight aggregates, not insulated	1	CO2data specifies that the product is ‘lightweight concrete blocks produced from expanded clay’.
Glass	Material located in *balconies_railing* subpart.	Laminated glass	1	
Mineral wool (hard)	The only type of hard mineral wood available in CO2data.	Glass wool insulation, density 100 kg/m3	1	
Mineral wool (loose) [density]	The MI dataset assumes mineral wool to be glass wool, distinguishing different densities (15/30/60 kg/m^3^).	Glass wool insulation, density 60 kg/m3 (CO2data’s GWP coefficients for glass wool insulation are the same regardless of the density, so any of them can be used)	1	Mineral wool could also be stone wool. For a given thermal conductivity, compared to glass wool, stone wool has approximately twice the density and half the embodied carbon per kg Thus, for a unit of volume the embodied carbon is roughly equal.
Mortar	Masonry mortar assumed as the most common type of mortar to alternatives with higher cement contents (frost masonry /general /plastering mortar).	Masonry mortar	1/2 (archetype-dependent)	Underestimates embodied carbon of other mortar types. If the user knows the mortar type in their case, they can adjust the coefficient.
Plasterboard	Material located in *ext.walls.*	Gypsum plasterboard, wind shield	1	
	Material located in *int.walls.*	Gypsum plasterboard, interiors	1	
Polystyrene insulation [density]	EPS assumed as the most common type of polystyrene insulation to the newer alternative, XPS.	EPS insulation	1 / 2	The dataset distinguishes 5 different densities (15/17.5/20/22/26), since the amount of embodied carbon is based on material mass. Using EPS emission figures slightly overestimates XPS emissions. If the user knows the polystyrene in their case is XPS, they can adjust the coefficient.
Steel	Material located in *chimneys* when concrete is not present or in *roof_covering*.	Colour coated steel sheet profile	1	
	Material located in brick ties, found in *ext.walls_non-bearing.layers.*	See Table 5	1 / 2 / 3 / 4 (see Table 5)	
	Material located everywhere else.	Steel rebar for concrete reinforcement	1 (for rebar) / 2 (for structural steel profiles)	Underestimates the embodied carbon of structural steel profiles.
Steel (stainless)		Stainless steel rebar	1	In the current MI dataset, all stainless steel in building structure is rebar.
Wood (solid)	Sawn timber known as the most common type of wood in structures.	Sawn timber	1 (for sawn timber) / 2 (for logs)	Slightly underestimates embodied carbon for logs.
Woodchip insulation	Woodchip insulation not available in CO2data. Used as the closest match, as woodchips are a by-product of sawn timber.	Sawn timber (density adjusted)	2	Historic product.
Wood fibre insulation (solid)	No dataset for wood fibre insulation without impregnation in CO2data. A board material used instead.	Fibreboard, soft, impregnated	1 (for impregnated fibreboards) / 2 (for non-impregnated fibreboards)	
Wood products	The MI dataset does not detail the type of wood product. Chipboard used as the best general proxy to alternatives (plywood).	Chipboard	1 (for chipboard) / 2 (for other board types)	Slightly underestimates embodied carbon for plywood.

Notes: In *‘*Match quality*’*, 1=accurate match (same material, same product), 2=best available match (same material, different product), 3=best available proxy (different material, same product), 4=possible proxy (different material, different product).

**Table 3:** Material matching between the complementary materials (windows and doors) in the MI dataset [4] and CO2data [3] for all materials present in dataset [2].

MI dataset material	Material matching criteria	CO2data material match	Match quality	Notes
Aluminium	Used in post-1990s building archetypes.	Aluminium profile, scrap 100%	1 / 2 (depending on the scrap content)	The only scrap contents available in CO2data. The scrap content has evolved over time; it has become increasingly common to utilise factory scrap and purchased scrap in the production of aluminium. Scrap contents’ temporal division based on source [12]. May under/overestimate carbon emissions if the actual scrap content is different.
	Used in 1990s building archetypes.	Aluminium profile, scrap min. 75%	1 / 2 (depending on the scrap content)	
	Used in pre-1990s building archetypes.	Aluminium profile, -tube or -rod, extruded, scrap 0%	1 / 2 (depending on the scrap content)	
Glass	The MI dataset does not detail the type of glass (float/coated/laminated/ insulating glass unit); float glass used as the best general proxy.	Float glass	1 (for float glass) / 2 (for other glass types)	Underestimates embodied carbon for glass types other than float glass.
MDF		Fibreboard, medium density, mdf, melamine coated	1	Only found in doors from the 1970s and onwards.
Mineral wool (hard)		Glass wool insulation, density 100 kg/m3	1	Only found in doors.
Steel	Steel in windows and doors (hinges, handles, etc.) assumed to be coated.	Metal coated light-weight steel profile or grill	1	
Wood (solid)	Wood in windows and doors assumed to be planed.	Sawn timber, planed	1	
Wood products	The MI dataset does detail the type of wood product. Chipboard used as the best general proxy to the alternative (plywood).	Chipboard	1 (for 1970s and newer doors) / 2 (for 1940s-60 s doors)	Only found in doors.

Notes: In *‘*Match quality*’*, 1=accurate match (same material, same product), 2=best available match (same material, different product), 3=best available proxy (different material, same product), 4=possible proxy (different material, different product).

CO2data [3] contains 304 products, processes, services, and systems. The product category, which is relevant for material matching, contains a total of 240 items that are divided into 10 groups. Groups contain from 15 products (‘building boards’ group) to 123 products (‘concrete’ group). The concrete group, for example, includes materials like ready-mix concrete, as well as composite products, like precast concrete elements that include steel reinforcement. Because the MI dataset [4] was created on a material level, material related, rather than composite, GWP coefficients were drawn from CO2data [3]. For example, ready-made coefficients for reinforced concrete structures were not used, but the constituent materials of the structures, steel reinforcement and concrete, were matched separately.

The version of the MI dataset [4] used to form the current embodied carbon dataset contains 22 materials (Table 2, Table 3, first column). Matching the materials with GWP coefficients from CO2data often depends on the material’s location the building, which is information provided by the MI dataset. First, this concerned whether the material is part of main building structures (Table 2) or complementary components, i.e. windows and doors (Table 3). Second, it concerned the more specific location in the parts or subparts of the structure, like being part of exterior walls, interior walls, floors, roofs, etc. (Table 2, Table 3, second column).

After considering the location, most of the material matches were straightforward: the material listed in CO2data [3] was found to match the material in the MI dataset [4]. Moreover, some materials did not have perfectly accurate matches or feasible alternatives in [3], so the closest option was considered as a sufficient proxy. Lastly, certain materials, such as mortar and glass, were recorded in the MI dataset [4] as such, even though there are more specific types of mortar and glass available in CO2data [3]. In these cases, the most basic type of the given material was selected as the most representative option. An assessment of the match quality is presented in the fourth column of Table 2, Table 3. Low-quality matches are compared with alternatives in the supplementary file. This is described further in the ‘Quality Control’ subsection.

### 4.3 Material stratification

After consulting CO2data’s [3] GWP coefficients for concrete and steel, further material stratification was necessary to find justified matches. The GWP coefficient of concrete varies depending on the strength grade, frost resistance (air-entrainment), and cement type (conventional or low-carbon). The difference between the GWP coefficients of the least and most carbon intensive strength grade and frost resistance combinations for conventional Portland-cement based concrete in CO2data [3], is 100% (0.1 kgCO_2_-eq/kg for ‘Ready-mix concrete, C20/25’ vs. 0.2 kgCO_2_-eq/kg for ‘Ready-mix concrete, C50/60, air-entrained’). In the case of steel, the difference between the GWP coefficients for corrodible and stainless steel is nearly sevenfold (0.67 kgCO_2_-eq/kg for ‘Steel rebar for concrete reinforcement’ vs. 4.6 kgCO_2_-eq/kg for ‘Stainless steel rebar’). Since a major proportion of a building’s embodied carbon is from reinforced concrete, it was considered crucial to estimate these materials with sufficient accuracy. As an example, concrete and steel account for 46–90% of the estimated embodied carbon in block of flats archetypes with concrete frames and facades.

Concrete and steel grades vary across cohorts and placements in buildings (here, *subparts*). The grade stratifications were based on literature like [[9], [10], [11]] to as great a degree as possible and supplemented with expert opinion when the information was not available in literature. The stratifications and sources are presented in the dataset sheet *reference_grades*. Most concrete grades identified by the stratification were available in the CO2data dataset [3], as shown in Table 4.

**Table 4:** Material matching between concrete grades stratified in [2] and materials available in or contrived from CO2data [3].

Concrete grade	Material matching criteria	CO2data material match	Match quality	Notes
C16/20	C16/20 is not available in CO2data, so C20/25 is used as the closest substitute.	Ready-mix concrete, C20/25	2	
C20/25		Ready-mix concrete, C20/25	1	
C20/25 *fr*	An air-entrained variant for grade C20/25 is not available in CO2data.	Ready-mix concrete, C20/25 air-entrained, modified (see notes)	2	The air-entrained GWP value was reconstructed by adding 0.3 to the base grade GWP, as the difference between recorded concrete grades and their corresponding air-entrained variants was typically 0.3.
C25/30		Ready-mix concrete, C25/30	1	
C30/37		Ready-mix concrete, C30/37	1	
C30/40 *fr*		Ready-mix concrete, C30/37, air-entrained	2	
C35/45		Ready-mix concrete, C35/45	1	
C35/45 *fr*		Ready-mix concrete, C35/45, air-entrained	1	
C40/50		Ready-mix concrete, C40/50	1	
Concrete in pre-1960s archetypes	No grade corresponding to the current grading scheme, but tests of samples indicate that the grade would correlate to C16/20 or C20/25 [11]. C16/20 not available in CO2data, so C20/25 is used.	Ready-mix concrete, C20/25	2	Before 1954, the building code did not specify concrete grades in the SI system currently used but gave the ratio of water:stone:cement in mixing concrete. Additionally, post-WWII deficits complicated access to concrete ingredients [11], so there is variation in the concrete used in these archetypes.

Notes: In *‘*Match quality*’*, 1=accurate match (same material, same product), 2=best available match (same material, different product), 3=best available proxy (different material, same product), 4=possible proxy (different material, different product). *fr* refers to ‘frost resistant’ or ‘air-entrained’ concrete mixtures.

In the MI dataset [4], brick ties that are not stainless steel were recorded as steel, even if other materials had been used. The tie materials used across decades and in different housing types differ as per Table 5, along with the CO2data [3] material match. The volume of brick ties in an archetype is minimal, resulting in a negligible impact on estimated embodied carbon. For example, in building archetype *BOF_CB_1990_1*, <1% of the building’s steel is in brick ties and alternative brick tie material choices resulted in a 1.7% change in an archetype’s embodied carbon intensity. No literature on brick ties was identified, so the materials used were approximated through expert opinion (Dr. Jukka Lahdensivu, personal communication, September 4, 2025).

**Table 5:** Material matching between brick tie material grades stratified in [2] and matched available in CO2data [3] used across archetypes.

Brick tie type	Material matching criteria	CO2data material match	Match quality	Notes
Brass wire	Assumed as the material found in building archetypes *BOF_CB_1960, BOF_CB_1970, ODH_CB_1960, ODH_BB_1960, ODH_BB_1970, ODH_WB_1950.*	Copper wire	3	Brass consists of copper and zinc, with copper in the majority.
Galvanised steel bar	Assumed as the material found in building archetypes *BOF_CB_1980, BOF_CB_1990, ODH_CB_1980, ODH_CB_1990, ODH_BB_1980, ODH_BB_1990.*	Metal coated light-weight steel profile or grill	1	
Galvanised nail	Assumed as the material found in building archetypes *ODH_WB_1960, ODH_WB_1970.*	Metal coated light-weight steel profile or grill	1	
Plastic tie	Assumed as the material found in the following archetypes *ODH_WB_1980, ODH_WB_1990.*	Water vapor barrier, PE	4	The type of plastic used in 1980s and 1990s plastic ties is not known to the authors.
Aluminium tie	Assumed as the material found in *ODH_CB_1970.*	Aluminium profile, -tube or -rod, extruded, scrap 0%	1	
Stainless steel tie	Assumed as the material found in all archetypes since 2000.	Stainless steel rebar	1	

Notes: In *‘*Match quality*’*, 1=accurate match (same material, same product), 2=best available match (same material, different product), 3=best available proxy (different material, same product), 4=possible proxy (different material, different product).

### 4.4 Estimating embodied carbon emissions

To calculate embodied carbon, a material’s volume and density were multiplied by its Conservative GWP coefficient. This process was followed for each subpart of each archetype, as described in Eq. (1):

(1) $$EC_{m,sp}=\;V_{m,sp}\times \;\rho_{m,sp}\;\times \;GWP_{m}$$

where *EC_m,sp_* (kgCO_2_-eq) is the embodied carbon of material *m* in building subpart *sp. V_m,sp_* (m^3^) is the volume of material *m* in building subpart *sp*. ρ*_m,sp_* (kg/m^3^) is the density of material *m* in the building subpart *sp. GWP_m_* (kgCO_2_-eq/kg) is the conservative GWP coefficient of material *m*.

The volumes of materials were retrieved from the MI dataset [4]. The densities were drawn primarily from the CO2data dataset [3] and as needed other sources (see *reference_co2data* in [2]). Respective GWP coefficients were identified from the CO2data dataset [3].

To determine the embodied carbon intensity of a subpart, part, level, or building, all material-specific embodied carbon values at the respective level of aggregation were summed and then divided by floor area, as described in Eq. (2):

(2) $$ECI=\;\frac{EC_{m_{1}}+EC_{m_{2}\;}+\ldots \;+EC_{m_{n}}}{FA}$$

where ECI (kgCO_2_-eq/m^2^) is the embodied carbon intensity for a subpart, part, level, or building. *EC_m1_* (kgCO_2_-eq) is the embodied carbon of material 1, *EC_m2_* is the embodied carbon of material 2, and so forth until material n, covering all materials within the subpart, part, level or building. *FA* (m^2^) is the floor area of the storey (when calculating ECI for a subpart, part, or level) or the entire building. Either GFA or TFA can be used for the type of floor area. The dataset [2] presents both *ECI_GFA_* and *ECI_TFA_*.

### 4.5 Quality control

Quality control regarding obtaining and recording the underlying material volumes data are described in [5], and the resulting material intensity coefficients (MICs) further are validated against other published MICs in [13]. The creation of the emissions dataset CO2data [3], in addition to its status as the official national source, is described in [7] and [14]. Therefore, this section focuses on the processes involved in combining these two data sources to produce [2]. An assessment of the material match quality is already provided in the Table 2, Table 3, Table 4, Table 5 for each material, and alternative matches are examined in the supplementary file.

Firstly, to ensure records are consistent between aggregation levels, sums of each individual material’s embodied carbon (*e_s_[material]* and *e_c_[material]*) were compared between all four data sheets. The embodied carbon values are calculated within those sheets, so referencing emissions factors from another sheet also revalidates the aggregation of material volumes (*v_s_[material]* and *v_c_[material]*) already conducted for [4].

Secondly, to check for potential input or calculation errors, building scale TFA-based embodied carbon intensities for each material (e.g. *ei_s_tfa_concrete*) were plotted and visually inspected. Embodied carbon intensities were used instead of absolute embodied carbon values to minimise the impact of building size. TFA was used because GFA is dependent on the program of spaces and could have thus introduced irrelevant variation. To account for inherent differences between different building types and main material choices, the comparisons were conducted separately for one dwelling houses and blocks of flats as well as each combination of bearing and façade material. Within these categories, per material values were expected to exhibit either a relatively even spread, indicating simple variety among the archetypes, or a clear dichotomy, indicating specific structural choices (e.g. whether a wood-framed building has a roof from sheet metal or not). Furthermore, certain materials were also considered in relation to one another: if a building had high embodied carbon intensity values for polystyrene and low mineral wool, that indicated specific insulation choices. The aforementioned process was also conducted for material volumes per square meter of TFA to account for coinciding material volume and emissions errors masking one another: for example, an erroneously high volume and low emissions coefficient might cancel each other out in the resulting embodied carbon intensity value. No clear outliers, pointing to random errors, were detected. All notable (but reasonable) deviations were checked and confirmed to correspond to archetype-specific properties such as structural choices.

Thirdly, following the same reasoning, the distribution between subparts in each archetype was plotted and visually inspected, again using TFA-based values, for both material volumes and material specific embodied carbon. Within a subpart, values were expected to either be approximately similar across all archetypes, e.g. the volume of materials or amount of embodied carbon in doors; similar across archetypes of the same kind, e.g. materials and embodied carbon in exterior walls’ bearing layers; or clustered based on structural choices, e.g. materials and embodied carbon in roof covering. As with the above examination, no clear outliers or unexplainable deviations were detected.

To address low-quality material matches, weak material matches were substituted for alternative materials listed in CO2data [3]. The supplementary file includes building-scale embodied carbon intensity comparisons between the selected materials from CO2data [3] and other available options. The comparisons reveal the magnitude of the impact of material matching on the embodied carbon at the scale of the whole building. If alternative material products were used, the greatest estimated decrease in intensity would be a 6.16% reduction and the greatest increase 18.59%. The latter is witnessed in the massive brick archetype *BOF_B_1950_1*, due to the high proportion of a single material. The least impactful material substitution is displayed among brick ties, due to their limited volume in the whole building.

The building scale reconstructed embodied carbon intensities were compared to reference values published in two reports commissioned by the Finnish Ministry of the Environment [15,16]. The reports provide intensities based on net heated area (NHA) and GFA, so the values from the dataset [2] also are recorded per square meter of GFA. In the dataset [2], the embodied carbon intensity for one dwelling houses ranges 97–172 kgCO_2_-eq/m^2^GFA for wooden houses, 175–241 kgCO_2_-eq/m^2^GFA for brick houses and 157–349 kgCO_2_-eq/m^2^GFA for concrete houses, depending on the construction decade and façade material. For blocks of flats, the intensities range 184–244 kgCO_2_-eq/m^2^GFA for concrete buildings. There is only one block of flats archetype that does not have a concrete bearing structure: *BOF_B_1950_1* has massive brick walls and a embodied carbon intensity of 230 kgCO_2_-eq/m^2^GFA. Wooden houses exhibit the lowest range, concrete buildings the highest, and brick buildings sit in between, which could be expected. The reference value reports [15,16] only provide module A1–A3 embodied carbon intensities for concrete row houses and blocks of flats. The reported ranges are 236–285 for row houses and 268–287 kgCO_2_-eq/m^2^GFA for blocks of flats [15], and 330–354 and 377–386 kgCO_2_-eq/m^2^GFA^1^ [16], respectively. The reports include MEP components in the scope, though [15] notes there may be inconsistencies in the underlying data regarding the MEP components and foundations. Alas, the reports do not provide embodied carbon intensity reference values for residential buildings constructed with materials other than concrete.

The embodied carbon intensities for the concrete structure archetypes in the dataset [2] cover the published reference value ranges [15,16] but also contain lower and higher values. The archetype in the dataset [2] that most closely aligns with the type, structure and age of the buildings reported in [15,16], namely *BOF_CC_2010*, estimates 226 kgCO_2_-eq/m^2^GFA without MEP components. When a square meter-based approximation for MEP components from CO2data [3] is added (61 kgCO_2_-eq/m^2^), the number rises to 287 kgCO_2_-eq/m^2^GFA. The value falls within the range published for blocks of flats in [15]. The increase of the reference values by one-third between the reports [15,16] may be explained by different methodologies used. The 2021 report [15] relied on circa 50 calculations for row houses and 270 calculations for blocks of flats performed by benchmark programme participants. The 2024 report [16] drew circa 210 calculations for row houses and 1200 calculations for blocks of flats from the same source but further manipulated the data by changing GWP coefficients. The methodology used to produce the results of the current dataset [2] is therefore closer to that of the 2021 report [15]. The 2024 report [16] notes that the manipulated results became substantially higher than the original calculations acting as the input data. Some differences between [2] and [15,16] could also be explained by further elements omitted from this dataset’s [2] building archetypes, like stairs and elevators, and context-specific features, like foundation piles and structural parking.

## Limitations

Because the GWP coefficients are contemporary, they represent embodied carbon associated with reconstructing similar buildings today, not historic production emissions. Presently, the Finnish energy grid and thus, the energy consumed in material production, is less emission-intensive than historically. Additionally, manufacturing processes and supply chains have evolved, and logistics have globalised. However, to the authors’ knowledge, no data on historic GWP coefficients are readily available in the Finnish context or elsewhere. Therefore, the used present-day GWP coefficients are the best available estimates. Furthermore, since some materials did not fully align between the MI dataset [4] and the Finnish national emissions dataset [3], the matching that the authors conducted had a subjective component.

The dataset excludes certain complementary building parts, like MEP components, stairs, and elevators, due to lack of sufficiently detailed information in case documents underlying [4]. Such components introduce additional embodied carbon content that is not accounted for in the dataset. Other exclusions include LCA modules beyond A3, such as transportation to the construction site (A4 [6]) and installation at the construction site (A5 [6]). Moreover, because the dataset relies on basic materials rather than composite products, it omits the product manufacture emissions and thus underestimates composite product emissions.

## CRediT Author Statement

**Chloe Kiernicki:** Data curation, Investigation, Writing – Original Draft, Validation. **Mario Kolkwitz:** Data curation, Investigation. **Tapio Kaasalainen:** Data curation, Writing – Review & Editing, Validation. **Satu Huuhka:** Conceptualization, Supervision, Writing – Original Draft, Writing – Review & Editing, Validation.

## Ethics Statement

The authors have read and follow the ethical requirements for publication in Data in Brief and confirm that the current work does not involve human subjects, animal experiments, or any data collected from social media platforms.

## Data Availability

ZenodoBuilding part specific embodied carbon dataset for residential buildings in Finland (Original data)

## Appendix Supplementary materials

Image, application 1

## Footnotes

1 The values reported in [16] have the denominator of m^2^NHA. The ranges above have been converted to m^2^GFA using the NHA and GFA values provided in Appendix 1 of the report [16].
